# High resolution diffusion tensor imaging of the human cortex reveals non-linear trajectories over the healthy lifespan

**DOI:** 10.1162/IMAG.a.115

**Published:** 2025-08-20

**Authors:** J. Alejandro Acosta-Franco, Graham Little, Christian Beaulieu

**Affiliations:** Department of Biomedical Engineering, University of Alberta, Edmonton, Alberta, Canada; Department of Computer Science, Université de Sherbrooke, Sherbrooke, Quebec, Canada; Department of Radiology and Diagnostic Imaging, University of Alberta, Edmonton, Alberta, Canada

**Keywords:** diffusion tensor imaging (DTI), healthy lifespan, cortical microstructure, human brain cortex, cortical radiality

## Abstract

The human cortex undergoes significant macrostructural and microstructural changes across the lifespan, which can be assessed using high-resolution diffusion tensor imaging (DTI). In healthy individuals, diffusion is typically greater perpendicular to the cortical surface, aligning with neuronal bodies and apical dendrites. This study examined DTI metrics in 190 healthy individuals (ages 5–74 years) to characterize normative cortical changes across neurodevelopment and aging. Whole-brain DTI data were acquired with 1.5 mm isotropic resolution and a b-value of 1000 s/mm² acquired in only 3:36 minutes at 3T. Cortical segmentation was performed exclusively on diffusion images to yield thickness, radiality, fractional anisotropy (FA), mean (MD), axial (AD), and radial diffusivity (RD) in total cortex as well as five lobes and were compared versus age. Cortical thickness decreased exponentially which differed from the diffusion metric cross-sectional age trajectories. FA, MD, AD, and RD exhibited u-shaped trajectories reaching minimum values in adulthood (~20–40 years). In contrast, radiality showed a cubic pattern, declining in childhood, stabilizing from 20–55 years, then decreasing again after 55, with the largest early-life changes in the temporal and occipital lobes and later-life declines in the frontal and parietal lobes. Steeper childhood DTI changes may reflect increased myelination of tangential fibers, as well as the growth of neuronal axons, somata, and dendrites, while elderly changes likely indicate reduced cell body density and radius. This study provides a baseline for future research into neurodevelopment and neurodegenerative diseases across the lifespan.

## 1 Introduction

Cortical development and aging follow distinct anatomical patterns. Early cortical maturation is characterized by rapid thickening to support cognitive and motor development, followed by progressive thinning due to synaptic pruning during childhood and adolescence (Fjell et al., 2009; Kelly et al., 2024). In adulthood and old age, cortical thinning continues gradually, particularly in regions such as the prefrontal cortex, which correlates with cognitive decline (de Chastelaine et al., 2019; Fjell et al., 2009; Zhou et al., 2013). However, cortical microstructure, which comprises not only neuronal cell bodies, apical dendrites, and axons, but also glial cells and vascular networks, presents different changes that are not defined by patterns of cortical thickness.

Cortical microstructure is organized predominantly in a mini-columnar structure that is perpendicular to the cortical surface (Gray & Lewis, 1918; Naidich et al., 2013). Diffusion tensor imaging (DTI) has been widely used to examine microstructural changes in the brain across the lifespan, primarily focusing on white matter tracts (e.g., Lebel et al., 2012). While white matter, with its organized and myelinated axonal structure, is well-suited for DTI analysis, the brain cortex also exhibits measurable diffusion anisotropy, although it poses unique challenges due to its complex folding and limited thickness (~1-5 mm in healthy adults). Despite these challenges, DTI studies have provided valuable insights into cortical microstructure. The cortex exhibits anisotropic diffusion properties that typically follow a radial pattern perpendicular to the surface, aligning with vertically oriented neural cell bodies and apical dendrites that extend from the white matter boundary (Jespersen et al., 2012; Kroenke, 2018; Thornton et al., 1997). High-resolution DTI studies (e.g., 0.94 mm isotropic and 1.14 x 1.14 x 3≈3.9 mm^3^) have shown that these properties are influenced by the columnar organization of the cortex (Heidemann et al., 2010; McNab et al., 2013), with large-caliber apical dendrites and unmyelinated tissue features being the primary determinant of cortical anisotropic diffusion, and myelination playing a secondary role (Assaf, 2019; Reveley et al., 2022). Radiality is a DTI metric exclusively from the cortex, and it plays a crucial role as a measure of diffusion alignment perpendicular to the cortical surface. It describes the angle of the primary diffusion direction of the tensor relative to the normal of the cortical surface (i.e., 0 parallel to surface to 1 perpendicular to surface) (McNab et al., 2013). Radiality can vary with cortical depth, and as shown by 0.9 mm isotropic and 1 mm isotropic studies, radiality shows higher values in intermediate layers (III-V) which are rich in pyramidal neurons with large-caliber apical dendrites (Kleinnijenhuis et al., 2015; Ma et al., 2023). These findings align with high-field DTI ex vivo studies using high resolution in humans (0.25 x 3 x 3 = 2.25 mm^3^, at 7T, and 0.3 x 0.3 x 0.3 ≈ 0.027 mm^3^, at 11.7T) and marmosets (0.15 x 0.15 x 0.15 ≈ 0.0034 mm^3^, at 7T), which revealed layer-specific diffusion disparities and nonlinear relationships between anisotropy and myelin content (Balasubramanian et al., 2021; Kleinnijenhuis et al., 2013; Reveley et al., 2024).

Cross sectional studies of cortical diffusion metrics as a function of age in humans have primarily been reported during early development. DTI studies in preterm neonates and infants have reported reductions of fractional anisotropy (FA) and mean diffusivity (MD) in the cortex during the firsts weeks of life, suggesting diminished radial cytoarchitecture, presumably associated with increased interneuron dendritic projections and synaptic growth (Ball et al., 2013; Eaton-Rosen et al., 2017; McKinstry et al., 2002; Ouyang et al., 2019; Smyser et al., 2016). The reduction in FA is driven by axial diffusivity (AD) decreasing more rapidly than radial diffusivity (RD), as indicated by a 1.2 mm isotropic DTI study in preterm infants (Monson et al., 2018). This period of rapid cortical maturation forms the foundation for cognitive and motor development. DTI trajectories, as seen in early development, remain similar during childhood and adolescence, where developmental remodeling processes are reflected in reductions in FA and all diffusivities, as shown in a high-resolution (1.5 mm isotropic) study of 621 healthy participants aged 8-21 years (Lynch et al., 2024).

Age-related changes in cortical diffusion properties in healthy adulthood and across the lifespan have been studied less extensively, mainly using low-resolution (≥2 mm isotropic) DTI protocols, revealing distinct cortical patterns with aging. Some cross-sectional DTI studies have reported increases of FA and diffusivities with age. A study in 72 healthy individuals (19-85 years) found a U-shaped FA trajectory with a minimum value at 36 years and linear increases in MD, AD, and RD (Lee et al., 2024). Similarly, a lobe-wise analysis in 85 healthy participants (15-55 years, only males) showed FA linear increases with a smaller effect in the temporal lobe and an MD increase in total cortex (Rathi et al., 2014). In contrast, a study exclusively done in visual cortex of 59 healthy volunteers (20–78 years) found no changes in FA, but U-shaped trajectories for MD, AD, and RD, with minima in mid 40s years of age (Malakshan et al., 2023). Some diffusion biophysical models, such as neurite orientation dispersion and density imaging (NOODI), in a cohort of 45 healthy adults (21–84 years) and 45 old people (65–85 years) showed neurite loss and reduced neurite density in widespread cortical regions with aging (Gozdas et al., 2021; Nazeri et al., 2015), while the soma and neurite density imaging (SANDI) model showed soma-related declines with aging in intra-soma signal fraction and soma radius (Lee et al., 2024). However, findings using lower resolutions have variations, as one study with 58 participants (21–83 years) using a 2 x 2 x 3 = 12 mm^3^ voxel size reported FA declines (but increase in diffusivities), potentially due to white matter contamination (Bouhrara et al., 2023).

By capturing the unique columnar and radial microstructure of the cortex, DTI has been shown to characterize developmental and age-related cortical changes. However, there are no DTI studies that show these changes across the lifespan, from early childhood to old age. The purpose here is to assess the typical cortical diffusion changes over the “lifespan” (5-74 years) in a single-site cross-sectional cohort of 190 healthy participants. This is achieved using a rapid 3:36 minutes, high-resolution (1.5 mm isotropic) whole brain DTI to yield whole brain and major lobe diffusion metrics measured by an automated cortical segmentation pipeline that works only on the diffusion images (Little & Beaulieu, 2021).

## 2 Materials and Methods

### 2.1 Participants and image acquisition

A cross-sectional cohort of 190 healthy control volunteers aged 5 to 74 years (108 female) participated in the study. Volunteers had no self-reported history of brain injury, neurological or psychiatric disorders. Recruitment occurred through advertising, and participants provided written informed consent, with underage volunteers obtaining both child assent and parent/guardian consent. Ethics approval was granted by the University of Alberta Human Research Ethics Board.

Whole-brain DTI data were acquired using a 3T Siemens Prisma with a 64-channel RF coil. A single-shot EPI protocol with Stejskal-Tanner gradients was used with the following: 90 axial/oblique slices at 1.5 mm thickness, 1.5 x 1.5 mm² in-plane resolution zero-fill interpolated on scanner to 0.75 x 0.75 mm², GRAPPA R=2 using a sum of squares method, phase partial Fourier 6/8, TR/TE = 4700/64 ms, 6 b = 0, and 30 b = 1000 s/mm² volumes and total acquisition time of 3:36 minutes.

### 2.2 Diffusion imaging preprocessing and segmentation

DTI images underwent preprocessing and cortex segmentation without the need for other structural images using a custom pipeline (Little & Beaulieu, 2021). Preprocessing involved correcting signal bias, which adjusts the non-central chi-squared signal distribution resulting from the sum of squares image reconstruction of the 64 channel phased array data to a Gaussian signal distribution (St-Jean et al., 2020). This reconstruction-induced positive bias especially affects regions with low signal-to-noise ratio (SNR), such as regions near the isocenter of the coil, and can lead to inaccuracies of diffusion metrics (e.g. low MD) if uncorrected. Correcting for this bias improves the accuracy of denoising, tensor fitting, and the resulting diffusion measures. Preprocessing also included non-local spatial and angular matching denoising (St-Jean et al., 2016), eddy current and motion correction (Andersson & Sotiropoulos, 2016), and utilization of Dipy v1.7.0 for tensor model fitting and DTI map generation (Garyfallidis et al., 2014). Additionally, a mean DWI b1000 was generated and corrected for intensity bias using N4 bias field correction, based on the spatial variations of signal intensity estimated from the first b0 image (Tustison et al., 2010).

**Fig. 1.**
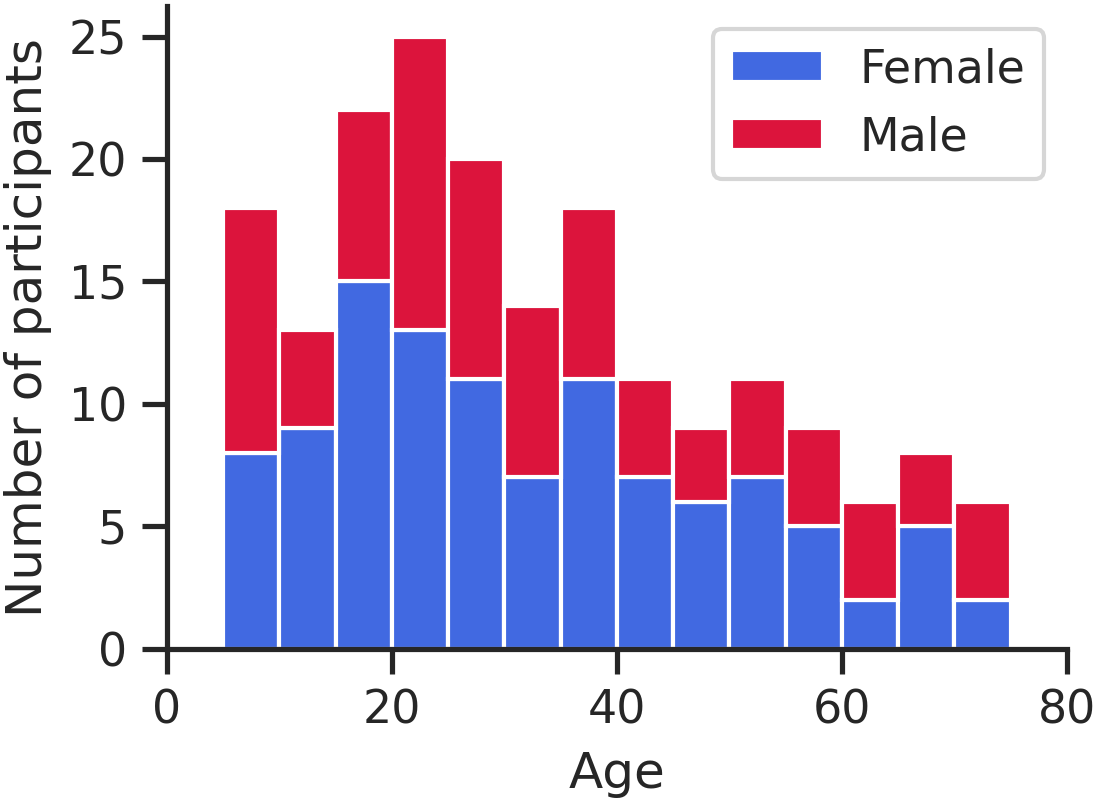
Histogram depicting the distribution of ages within the cohort, divided into bins of 5 years. The total cohort consists of 190 subjects, including 108 (57%) females and 82 (43%) males.

**Fig. 2.**
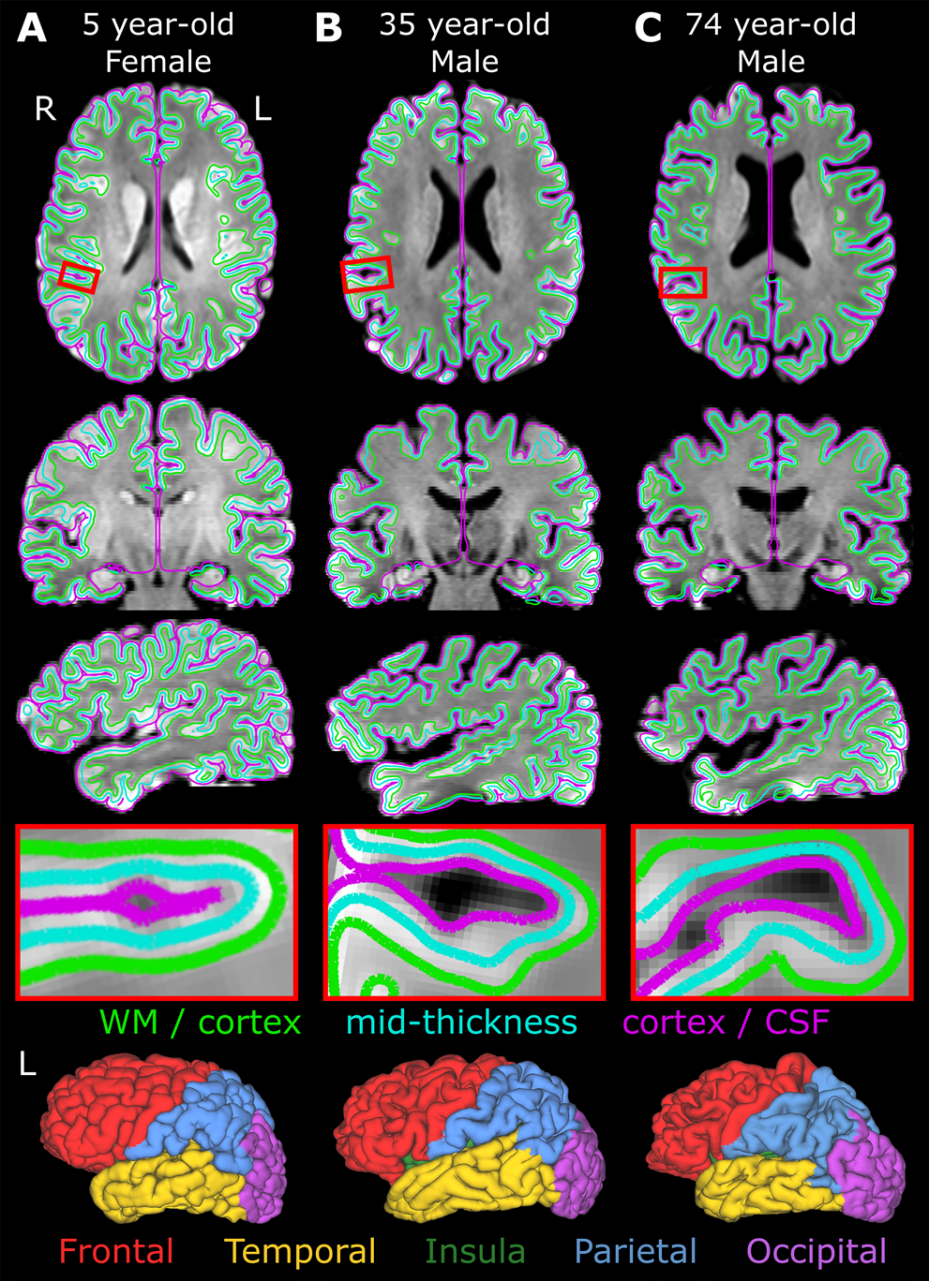
Examples of the diffusion image only automated segmentation to delineate the cortical surfaces (magenta - cortex/CSF, cyan - midthickness, and green - WM/cortex), as shown on the mean 1.5 mm isotropic DWI in three orthogonal planes for three participants (A–C) representing different ages across the lifespan. The red boxes show zoomed in regions. The bottom row shows their five lobe segmentations in the left hemisphere (cortex/CSF surface is shown).

The cortex segmentation algorithm only uses high-resolution diffusion images (mean b1000 DWI intensity and FA maps) instead of traditional T1 or T2-weighted images, as previously described (Little & Beaulieu, 2021). This outputs a 3D surface segmentation of the inner (WM/cortex) and outer (cortex/cerebrospinal fluid (CSF)) surfaces of the cortex as well as a mid-thickness surface located midway. The quality of segmentations was assessed via visual inspection, excluding 11 subjects from an initial group of 201 participants, resulting in a final cohort of 190 participants listed earlier. A histogram of the distribution of the participant ages within the cohort is shown in Figure 1. Brodmann’s areas, as mapped to a population average template (Van Essen et al., 2012), were combined to create a parcellation for each brain lobe: frontal, temporal, insula, parietal, and occipital, as described in the original publication of the segmentation pipeline (Little & Beaulieu, 2021). Figure 2 shows the accurate delineation of the three cortical surfaces that were created for this paper using only DWI data: cortex/CSF, mid-thickness and WM/cortex; as well as the five lobe segmentations in the native space in three example participants across the lifespan at 5, 35 and 74 years of age (see Supplementary Fig. 1 for example FA, MD, AD, and RD maps of these three participants).

### 2.3 Cortical thickness and diffusion measurements

**Fig. 3.**
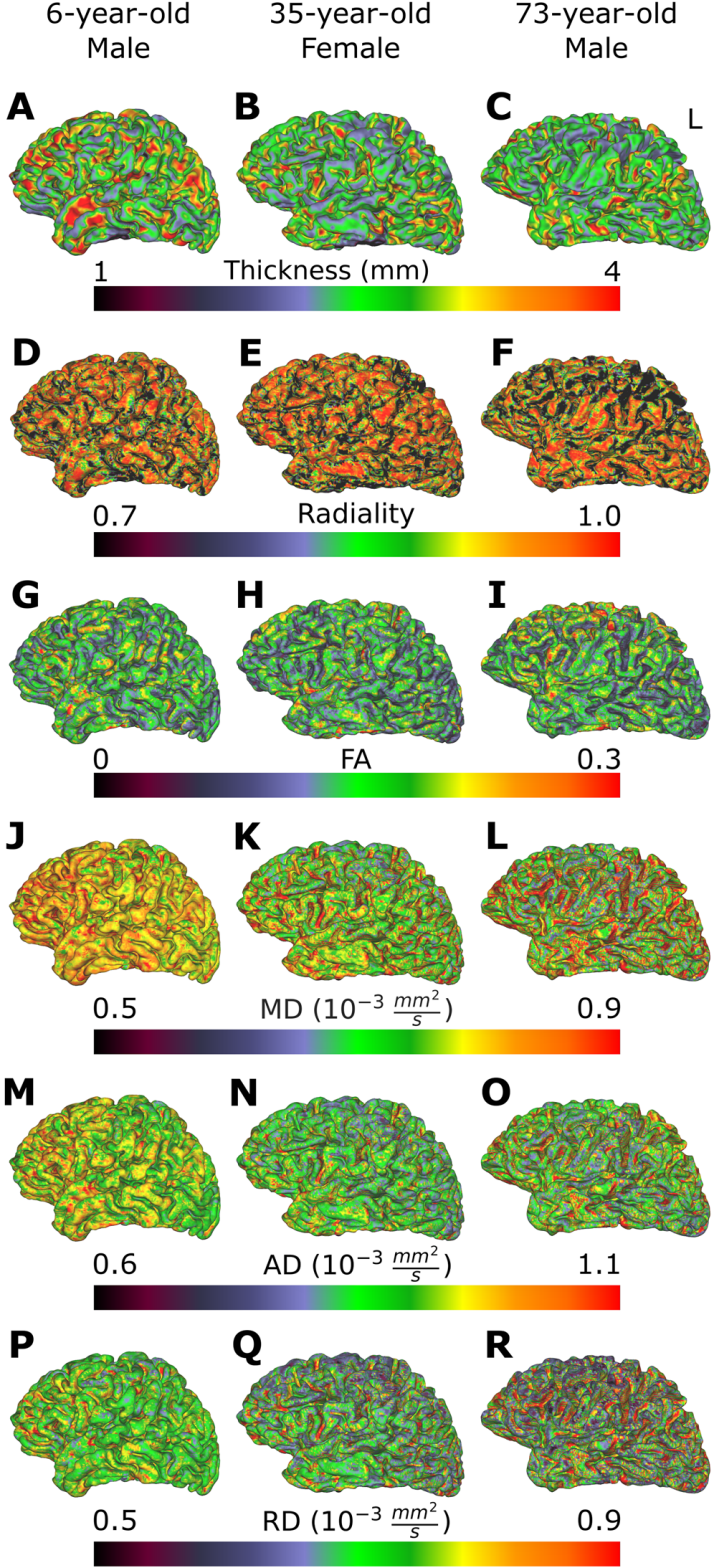
(A–C) Cortical thickness and (D–R) DTI metrics of radiality, FA, MD, AD, and RD are visualized on mid-thickness surfaces, showcasing three subjects across the lifespan: aged 6 years old, 35 years old and 73 years old.

Six cortical metrics were analysed, with five based on the tensor model, namely FA, MD, RD, AD and radiality (McNab et al., 2013), and cortical thickness. To reduce partial volume measurement from surrounding CSF and superficial WM, DTI metrics and primary eigenvectors (to compute radiality) were first sampled using nearest-neighbour interpolation onto each vertex of the mid-thickness surfaces. Figure 3 showcases mid-thickness cortical surfaces, with thickness and DTI metrics of three representative subjects across the lifespan at ages of 6, 35, and 73 years of age. In addition, vertices were eliminated with MD > 1.5 x 10^-3^ mm^2^/s or FA > 0.3, because values above those thresholds are outside the range of cortical tissue (Assaf, 2019; Lynch et al., 2024). Radiality is calculated for each vertex as the absolute value of the dot product of the orientation of the mid-thickness surface normal and the primary eigenvector interpolated for a given vertex. The cortical thickness was obtained by calculating the Euclidean distance (in mm) of each paired vertex between the inner and outer cortical surfaces (Makropoulos et al., 2018). Total cortex and lobe-wise measurements of all diffusion metrics were extracted separately for the left and right hemispheres of each subject. For further statistical analysis, the average of all cortical metrics was calculated for each region (total cortex and each lobe) per hemisphere.

### 2.4 Statistical analysis

Scipy library v1.5.4 was used for all the statistical testing (Virtanen et al., 2020). The paired Wilcoxon Rank Sum Test was used to test for hemisphere differences first and then the unpaired Mann-Whitney U Test was used to test for sex differences. As discussed in the Results, there were no statistical differences in hemisphere or sex comparisons for total/all five lobes separately and thus data from both hemispheres were averaged and males and females were combined into a single group to simplify all subsequent analyses.

Group differences of cortical metrics across the lobes were evaluated with one-way ANOVA followed by post-hoc Tukey’s test. All tests used false discovery rate with a corrected p-value <0.05 for significance. In addition, a median absolute difference group-wise was required to consider a difference significant: >0.1 mm for thickness, >0.03 for radiality, >0.01 for FA, >0.01 x 10^−3^ mm^2^/s for MD, AD and RD. Differences smaller than these values in DTI metrics can be considered negligible as in our previous white matter DTI lifespan study (Lebel et al., 2012). The same margins of error were shown in test-retest comparisons using high-resolution DTI to study the cortex (Little & Beaulieu, 2021), indicating that these margins are attributed to methodological imperfections rather than actual microstructural alterations.

Cross-sectional cortical data were analyzed using a set of five fitting equations: linear, quadratic, cubic, Poisson, and exponential decreasing, adjusted using ordinary least squares regression. These equations were selected because they describe multiple possible trajectories across the lifespan: a linear fit shows steady trajectories, quadratic and cubic fits show fluctuating trajectories, a Poisson fit shows non-symmetric curved trajectories while keeping a small number of parameters (Lebel et al., 2012), and an exponential fit decreasing shows decreasing trajectories with a steep drop at younger ages. The cortical data was fit for average values extracted from the entire cortex to depict general trajectories over the lifespan, and the fitting procedure was repeated for values extracted for each lobe. To select the best model for each metric and region of interest, the Akaike Information Criterion (AIC) values were compared among regression models. The model with the lowest AIC value is considered the best fit, but models with AIC values within two units of the lowest are still regarded as similarly accurate (Burnham & Anderson, 2002), and they are preferable if they use fewer parameters. The trajectories, peaks, and minimum values across the lifespan were described based on the selected models.

## 3 Results

Figure 2 shows the accurate delineation of the three cortical surfaces that were created using only DWI data: cortex/CSF, mid-thickness and WM/cortex; as well as the five lobe segmentations in the native space in three example participants across the lifespan at 5, 35 and 74 years of age. This expands the age span application of the cortex segmentation software, as it was originally published in a healthy adult cohort aged 20-38 years (Little & Beaulieu, 2021). Figure 3 showcases midthickness cortical surfaces, with thickness and DTI metrics of three representative subjects across the lifespan at ages of 6, 35, and 73 years of age. From these surfaces, the cortical thickness appears to get smaller with age across the total cortex (Fig. 3A-C), while diffusion metrics have distinct patterns. For instance, in the radiality surfaces (Fig. 3D-F), areas of larger values are mainly concentrated in the gyri in the three participants, although there are areas of lower values as seen particularly in the parietal lobe of the oldest participant. FA surfaces (Fig. 3G-I) show scattered areas of larger values in the youngest and oldest participant, with lower FA values overall in the middle-aged participant. Regarding the diffusivities MD (Fig. 3J–L), AD (Fig. 3M–O), and RD (Fig. 3P–R), the total cortex surfaces of the youngest participant show homogeneously large values overall, while the middle and older participants show smaller and larger diffusivity values in widespread areas, respectively.

### 3.1 Lobe group comparisons

No statistical differences were observed in hemisphere or sex comparisons for any cortical metric (data not shown), and thus all subsequent results are with left and right averaged per participant and males and females were combined into a single group.

**Fig. 4.**
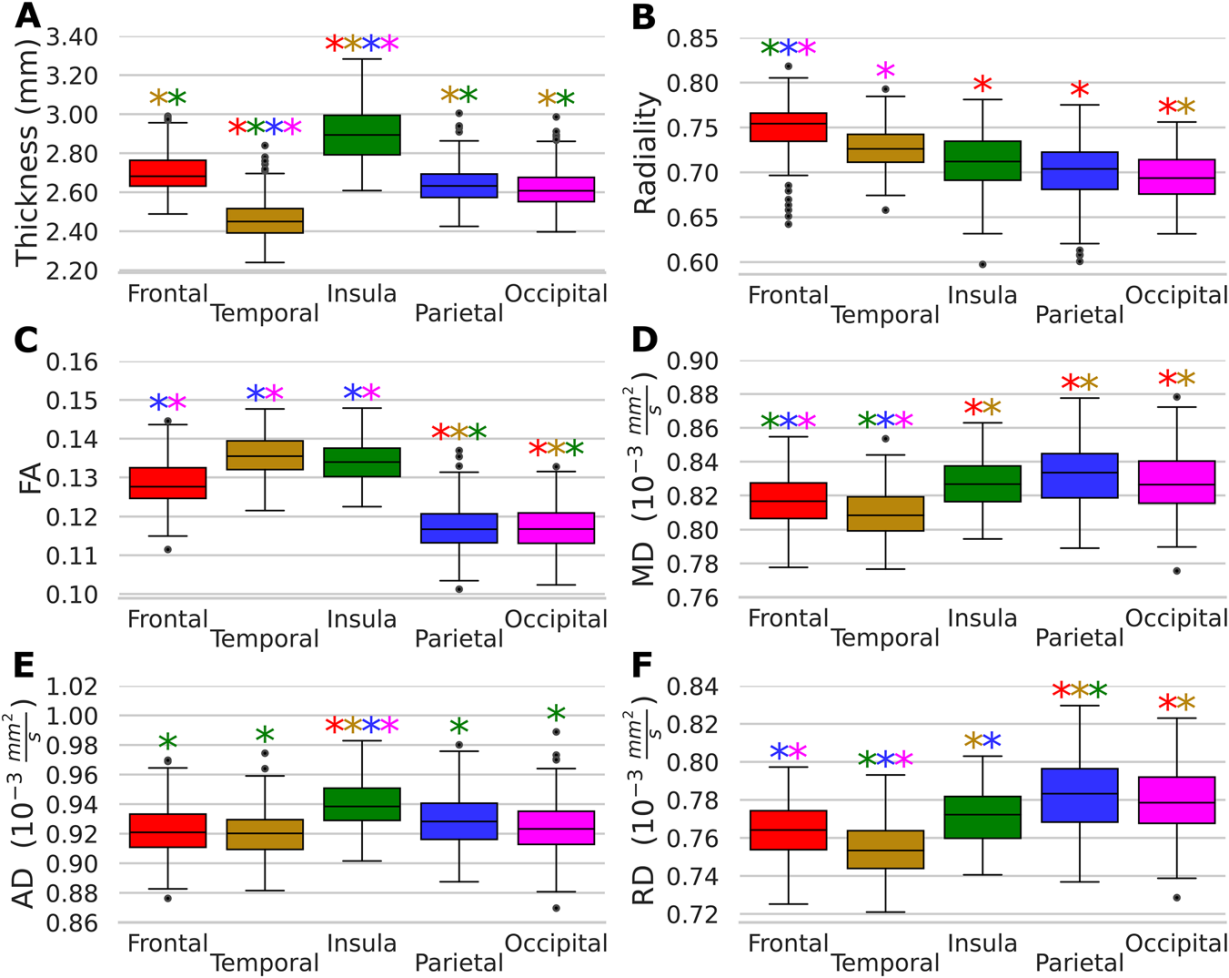
Inter-lobe comparisons of scalar measures across the cohort. Significant between-lobe differences (p < 0.05) are indicated by the color of the asterisk. Regions with higher values were (A) the insula for cortical thickness, (B) frontal lobe for radiality, (C) frontal/temporal/insula for FA, (D) insular/parietal/occipital for MD, (E) insula for AD, and (F) parietal/occipital for RD.

Over the entire cohort including all ages, ANOVA tests revealed significant differences in all six cortical metrics across lobes (Fig. 4), with post hoc Tukey’s test showing differences among all lobes in all cortical metrics (p < 0.001). The largest variations are described as follows in order of largest percent difference between lobes with maximum and minimum shown: (i) cortical thickness varies by 15.1% from insula (2.90 mm) to temporal (2.46 mm); (ii) FA varies by 13.8% from temporal (0.14) to parietal and occipital (0.12); (iii) radiality varies by 7.4% from frontal (0.75) to occipital (0.69); (iv) RD varies by 3.7% from parietal and occipital (0.78 x 10^-3^ mm²/s) to temporal (0.75 x 10^-3^ mm²/s); (v) MD varies by 2.6% from insula, parietal, and occipital (0.83 x 10^-3^ mm²/s) to temporal (0.81 x 10^-3^ mm²/s); and (vi) AD varies by 2.0% from insula (0.94 x 10^-3^ mm²/s) to temporal and frontal (0.92 x 10^-3^ mm²/s).

### 3.2 Whole brain cross sectional age trajectories

**Fig. 5.**
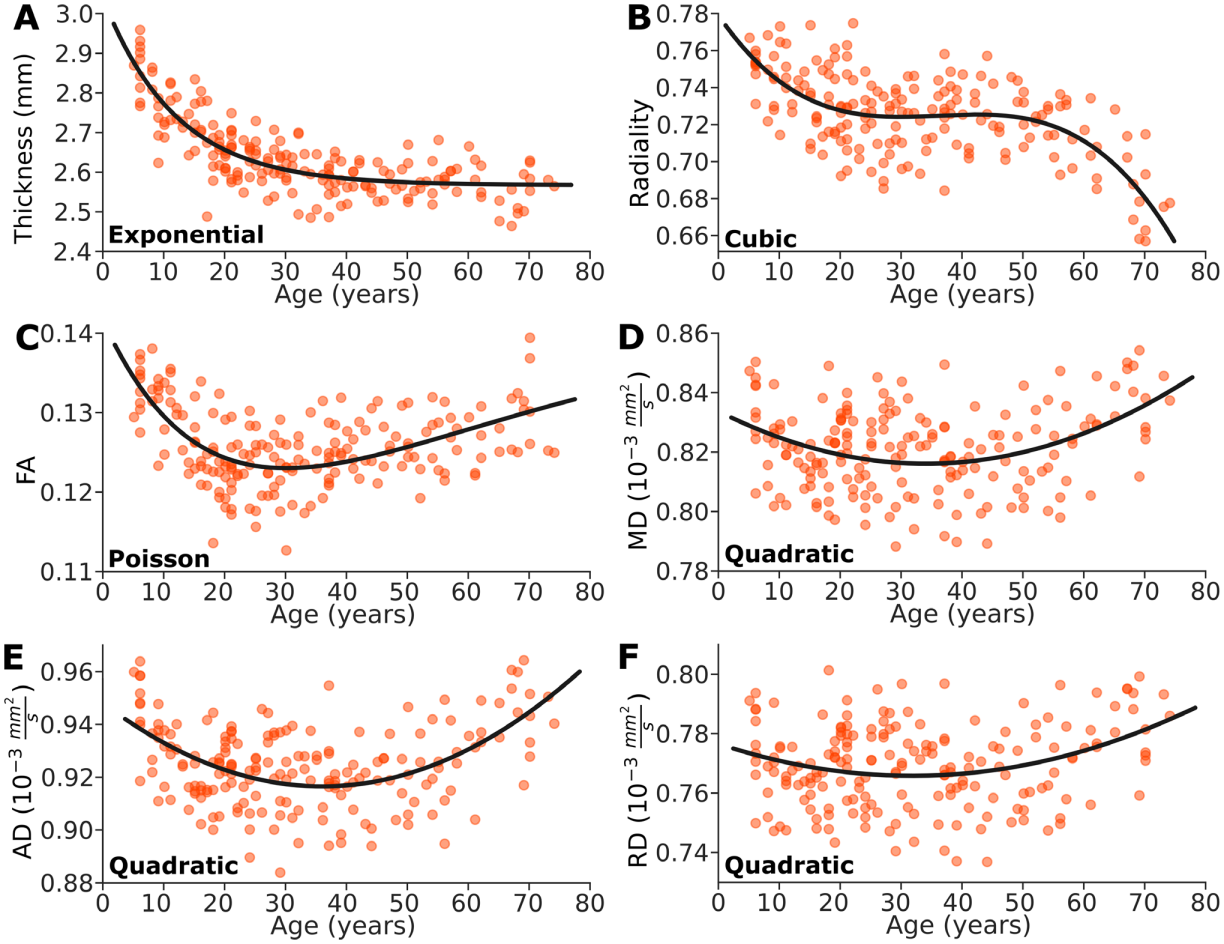
Developmental and aging trajectories of mean cortical metrics across the entire cortex are displayed, along with the best-fit model (black lines). (A) Thickness shows a steep drop in childhood with an exponential decreasing trajectory. (B) Radiality is the only metric that shows a cubic trajectory, with steeper drops <20 and >55 years. (C) FA shows a steep drop (note: not an increase like in white matter) until 28 years followed by a slow increase. (D–F) MD, AD and RD exhibit U-shaped quadratic trajectories with minimum values around 35 years of age. AD shows steeper changes than RD resulting in the FA reduction then increase.

DTI metrics in the total cortex revealed non-linear patterns throughout the lifespan. Cortical thickness (Fig. 5A) exhibited a decreasing exponential trajectory, with a steep decline from 2.87 mm at age 5 to 2.60 mm at age 30, followed by a minor gradual decrease up to age 74. Radiality (Fig. 5B) followed a cubic trajectory with steeper declines during childhood, from 0.76 at age 5 to 0.73 at age 19, remaining quasi-steady around 0.72 from age 20 to 54, and then undergoes another steep decline from 0.71 at age 55 to 0.65 at age 74. The distribution of cortical radiality was right-skewed, with a large proportion of vertices showing high values (radiality > 0.9), consistent with previous findings (Little & Beaulieu, 2021). This proportion decreased slightly with age, from 40% in the child/adolescent group to 37% in young/middle-aged adults and 33% in the oldest group. FA (Fig. 5C) followed an initially decreasing Poisson trajectory starting at 0.14 at age 5 and reaching a minimum of 0.12 at age 30 with a subsequent gradual increase through adulthood, nearly returning to childhood levels at 0.13 by age 74. All diffusivity values followed U-shaped quadratic trajectories, reaching their minimum values during adulthood: MD (Fig. 5D) of 0.82 x 10^-3^ mm²/s at age 34, AD (Fig. 5E) of 0.92 x 10^-3^ mm²/s at age 36, and RD (Fig. 5F) of 0.76 x 10^-3^ mm²/s at age 32. Notably, AD dropped to a greater extent and increased to a greater extent than RD (leading to the observed FA reduction in development and subsequent increase). The diffusivity metrics were slightly higher by ~0.01 x 10^-3^ mm^2^/s at older ages compared to the youngest ages: MD of 0.83 x 10^-3^ mm²/s at age 5 and 0.84 x 10^-3^ mm²/s at age 74, AD of 0.94 x 10^-3^ mm²/s at age 5 and 0.95 x 10^-3^ mm²/s at age 74, and RD of 0.77 x 10^-3^ mm²/s at age 5 and 0.78 x 10^-3^ mm²/s at age 74.

### 3.3 Lobe-wise cross-sectional age trajectories

**Fig. 6.**
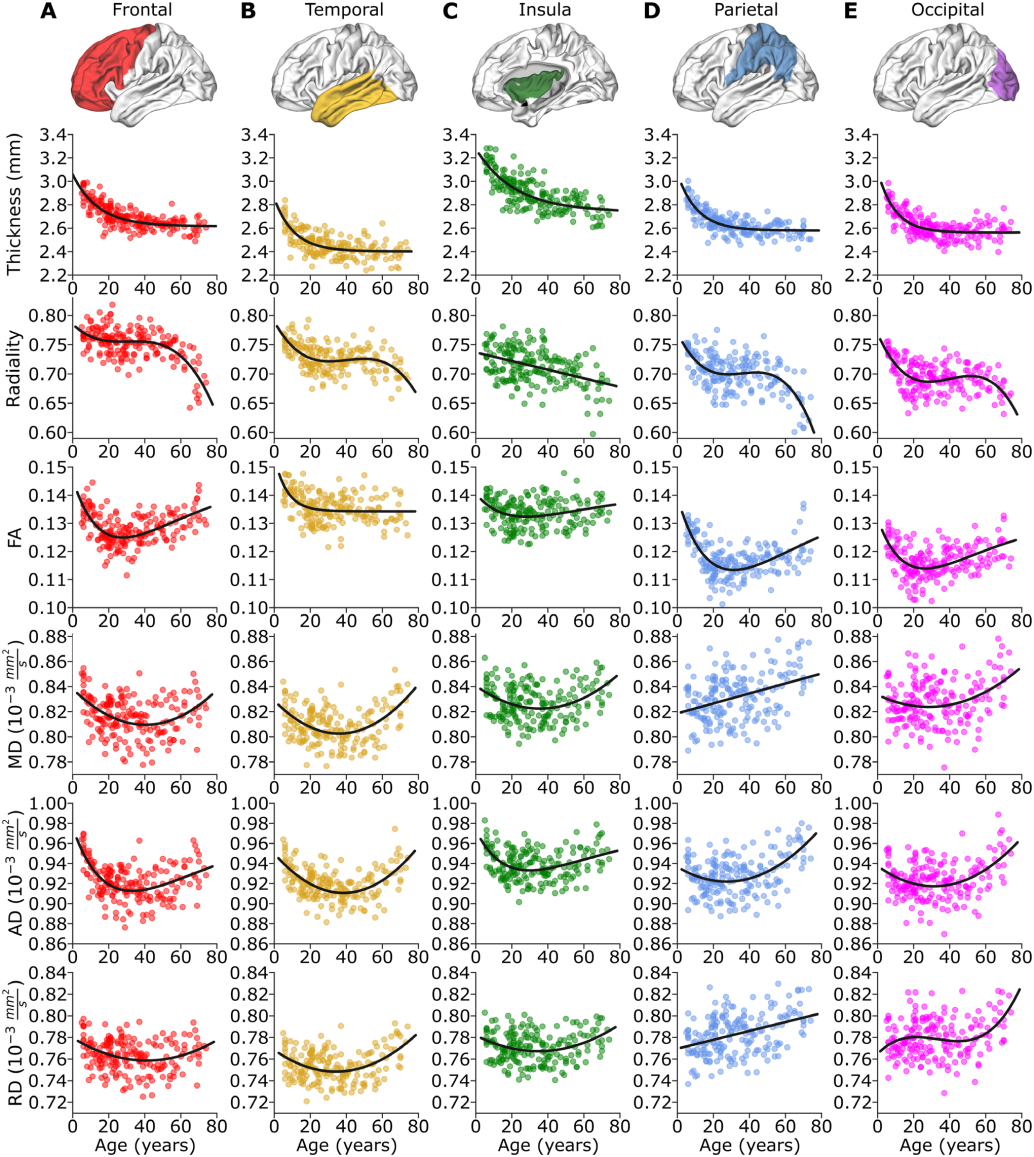
Each row displays data for all six cortical metrics across age within each of five lobes (A–E). Row 1: Thickness in all lobes showed an exponential reduction with age. Row 2: Radiality follows a cubic trajectory in 4/5 lobes with an initial drop in childhood/adolescence which then levels off then drops again markedly ~50-60 years. The insula shows a linear decrease with age. Row 3: FA follows an initial decrease followed by a subsequent increase in most lobes, except for the temporal lobe which flattens out after 20 years. For the other lobes, FA reaches its minimum value in the early/mid-20s. Rows 4-6: MD, AD, and RD mainly show down then up trajectories, where the minimum values are typically reached in the late 30s. AD gets smaller earlier and by a greater amount than RD, which leads to the FA reduction during neurodevelopment. Conversely, the AD goes up more than RD with aging towards the elderly, leading to elevations of FA. Yet in both cases, radiality drops in those two age spans.

For the most part, the age trajectories of the five individual lobes (Fig. 6) mirrored those observed in the total cortex. Cortical thickness displayed an exponential decrease across all lobes, with the insula, the thickest brain lobe, exhibiting a more prolonged and larger decrease of 0.41 mm (13%), from 3.17 mm at age 5 to 2.76 mm at age 74, while other lobes decreased around 0.30 mm (11%) up to age 30, followed by minimal further reductions until age 74.

Radiality showed cubic trajectories in 4/5 lobes whereas the insula displayed a decreasing linear trajectory, with a difference across 5–74 years of 7% (from 0.73 to 0.68). The frontal lobe radiality changed minimally from childhood from 5 to 20 years with a value of 0.76 which dropped slightly to ~0.75 and then was level until age 52, followed by a steep drop of 11% to 0.65 by age 74. Temporal, parietal, and occipital lobes also showed a plateau with changes less than 2% during most of youth and adulthood (ages 20 to 55). However, relative to the frontal lobe, these lobes had steeper radiality trajectories during childhood to young adulthood (~6%) and then underwent another reduction after age 55 with similarly large changes in the parietal lobe (12%), which were not as extensive in the temporal (4%) and occipital lobes (6%).

FA versus age is described by Poisson fits in 4/5 lobes with a steeper initial decrease, reaching minimum values in adulthood ~ age 26–28, and then going up more gradually without reaching the FA values in the youngest. The insula shows the smallest FA reduction (4%) from 5 to 26–28 years while the frontal, parietal, and occipital lobes change by 9%, 13%, and 8%, respectively. FA in the temporal lobe was the only lobe showing an exponential trajectory with most of the change (7%) occurring by age 20 and then was flat.

MD versus age showed down then up quadratic trajectories in frontal, temporal, insula, and occipital lobes with similar values at the beginning and end of the lifespan (difference of less than 1.2%). The age of minimum MD was the smallest for occipital at 28 years whereas it was 36–40 years for frontal, temporal, and insula. In contrast, the parietal lobe had an increasing linear trajectory for MD. The AD and RD also showed U-shaped trajectories mainly, where there was a greater and earlier decrease of AD than RD, which led to the lower FA with neurodevelopment into adulthood. With aging, AD went up by a greater extent than RD leading to the observed elevated FA into the elderly group. Notably, the parietal and occipital lobes showed AD and RD values at 74 years which exceeded that at 5 years.

## 4 Discussion

This study addresses a gap in the literature by using a high-resolution (1.5 mm isotropic whole brain) DTI protocol to examine cross-sectional trajectories of cortical diffusion metrics (radiality, FA, MD, AD, and RD) across a broad age range, from childhood (5 years) to old age (74 years). This approach enables the identification of developmental and aging-related diffusion trends that differed from cortical thickness. In contrast to DTI studies of white matter over the lifespan, cortical FA decreased during development and then increased with aging. Previous research over 8 to 21 years also showed that cortical FA decreased linearly, while MD, AD, and RD decreased non-linearly (Lynch et al., 2024). Similar patterns of cortical anisotropy decreases have been observed during neurodevelopment in animal models (e.g., mice, ferrets, and baboons), suggesting a transition from a highly organized radial cytoarchitecture to a more complex, isotropic neuronal arrangement as dendritic arborization and synaptic connectivity increase (Jespersen et al., 2012; Kroenke et al., 2005, 2007; Mori et al., 2001). Most of the diffusion studies conducted in adults and elderly agree with observations showing increases in diffusivities with age, while FA has shown the most disparity: either showing increase, decrease, or no change (Bouhrara et al., 2023; Lee et al., 2024; Lynch et al., 2024; Rathi et al., 2014). Only one study found a U-shaped quadratic trajectory of FA (with minimum at 36 years) (Lee et al., 2024), while other studies found quadratic U-shaped trajectories for diffusivities (with minimum around 40 years) (Bouhrara et al., 2023; Malakshan et al., 2023). Those previous cortical DTI studies have not covered an age range as broad as the present study, and all exclude childhood/adolescence (<15 years old), a period with a distinct DTI trajectory. As a result, these studies often show more linear trends in their analyses, potentially overlooking key inflection points, such as minimum values in age-related trajectories.

The inclusion of radiality in this study strengthens the characterization of DTI metrics across the lifespan beyond FA, MD, AD, and RD—radiality is reported here for the first time in vivo in a cross-sectional cohort covering a wide age range. The observed patterns in cortical diffusion metrics align with previous studies on white matter maturation and degeneration, which reported maximum FA and minimum MD around age 30 for most white matter tracts (Lebel et al., 2012; Westlye et al., 2010). These trajectories are opposite with those found in the cortex in this study, reinforcing the idea that cortical maturation follows a distinct timeline from white matter. This study provides foundational data on cortical diffusion properties across the lifespan, establishing a baseline for future research into cortical microstructure in aging and neurodegenerative diseases. Radiality emerged as a particularly interesting metric, revealing distinct patterns across the lifespan. It exhibited two steep declines: one during childhood/adolescence (5–19 years) and another in adulthood (55–74 years). These findings emphasize radiality’s potential role in characterizing the cortical columnar organization, as it does not exhibit strong correlations with other DTI metrics, suggesting it captures unique aspects of cortical microstructure (Heidemann et al., 2010; Kleinnijenhuis et al., 2013; McNab et al., 2013).

The decrease in cortical radiality across the lifespan is likely driven by complex microstructural remodeling. Early in life, the cortex is more radially organized, with perpendicularly aligned neurons. As dendritic arborization and intracortical connectivity increase throughout maturation, the architecture becomes more complex and less directionally coherent. This developmental remodeling introduces more tangential structures, reducing the predominance of radial diffusion patterns (McKinstry et al., 2002; Ouyang et al., 2019). The decline in radiality observed during aging likely reflects different underlying mechanisms. Aging is associated with dendritic retraction and a simplification of the apical dendritic arbors of pyramidal neurons (Dickstein et al., 2013). This reduction in dendritic complexity diminishes the directional coherence of water diffusion perpendicular to the cortical surface. Additionally, age-related loss of intracortical myelination may further reduce microstructural anisotropy by eliminating barriers that constrain water movement along radial trajectories (Grydeland et al., 2013). Together, these developmental and aging-related changes suggest that radiality captures distinct biological processes operating at different stages of life, both contributing to reductions in anisotropy aligned with the cortical columnar structure.

Different lobes exhibited distinct trajectory shapes for the same DTI metric, which likely reflects regional biological variability rather than modeling artifacts. Despite differences in the specific best-fit models, general patterns were consistent across lobes. For instance, FA tended to decrease from childhood to young adulthood and increase again in older age, while diffusivities followed U-shaped trajectories with minima near the third decade of life. These consistent inflection points suggest that the observed changes reflect meaningful cortical microstructural dynamics.

Regional variability in diffusion trajectories, including radiality, likely reflects underlying cytoarchitectonic differences such as cortical layering, myelin content, and dendritic architecture. Prior studies using T1w/T2w ratios as a proxy for intracortical myelin have shown substantial variability across the cortex: primary sensory and motor areas tend to have high T1w/T2w values (indicating greater myelin content), while regions like the temporal lobe and insula show consistently lower values (Glasser & Van Essen, 2011; Grydeland et al., 2019). Myelin levels tend to stabilize between the ages of 30 and 60 years (Grydeland et al., 2019), potentially influencing DTI metrics across the lifespan. Radiality, in particular, may be sensitive to such variations. Surface-based diffusion orientation analyses have revealed distinct radiality patterns between cytoarchitectonically distinct areas like the primary motor and somatosensory cortices, driven, in part, by differences in granular layering and apical dendrite structure (Balasubramanian et al., 2021; Little & Beaulieu, 2021; McNab et al., 2013). Moreover, the contribution of individual cortical layers to the DWI signal can be modulated by T2 relaxation times; layers with longer T2 values, such as the less-myelinated superficial layers, may contribute disproportionately to the measured signal (Reveley et al., 2022) further shaping regional diffusion profiles and radiality patterns.

The insula, in particular, exhibited a distinct diffusion pattern. While it had the thickest cortex (average 2.9 mm), it showed minimal change in FA across the lifespan (ranging from 0.13 to 0.14) and a linear decline in radiality. This might be attributed to the insula’s unique cytoarchitecture, which consists of both granular and agranular regions. The anterior insula shares characteristics with the prefrontal cortex due to its well-developed granular layers, while the posterior insula has an agranular structure like that of primary sensory areas (Bauernfeind et al., 2013). Additionally, the presence of specialized neuronal populations, such as Von Economo neurons and fork cells (Allman et al., 2010), may further account for the distinct diffusion properties observed in this region. While a few regional exceptions remain without a clear anatomical explanation, they may reflect finer-scale microstructural variability not captured by lobe-level analyses.

While this study employed high-resolution DTI with a 1.5 mm isotropic voxel size, which allowed for detailed measurement of cortical diffusion properties, certain limitations must be acknowledged. First, resolving fine-scale microstructural features, such as crossing fibers within the cortex, remains challenging at this resolution, as the signal from the six cortical layers is averaged. Partial volume effects from neighboring white matter and CSF could also introduce bias into diffusion measurements. One approach to mitigating these issues is increasing spatial resolution beyond 1.5 mm isotropic; however, this requires longer scan times for whole-brain coverage and custom pulse sequences (Haldar et al., 2020; Wang et al., 2021). Alternatively, advanced diffusion models, such as multi-tensor diffusion (Villaseñor et al., 2023), may provide better separation of microstructural components, complementing higher-resolution acquisitions. Additionally, protocols designed to suppress signal from adjacent tissues (Baron & Beaulieu, 2015; Concha et al., 2005) could further enhance specificity in cortical diffusion measurements.

This study did not distinguish between the cortex at the crowns of gyri and the deeper sulci, despite their differing neuronal organization. In gyri, neuronal somata and apical dendrites tend to align radially, whereas in sulci they are often compressed tangentially with wavy trajectories (Hilgetag & Barbas, 2005). Surface-based analysis also introduces sampling variability (only in radiality), particularly in regions with high curvature, where geometry (e.g., small changes in vertex orientation) can lead to wide ranges in radiality values sampled from the same voxel. While the use of five major lobar parcellations helped to establish broad lifespan trends, the surface averaging across lobes in this study may have overlooked these distinctions. Future work will benefit from incorporating finer parcellation schemes, such as those from the Human Connectome Project (Glasser et al., 2016), and from curvature-based analyses to more accurately capture spatial specificity and improve interpretation of radiality and other DTI-derived metrics across the cortex.

## 5 Conclusions

This study provides critical insights into the trajectories of cortical diffusion metrics across the lifespan, from childhood through old age. The results indicate that cortical microstructure undergoes unique changes over time, with diffusion metrics exhibiting complex, non-linear patterns that reflect underlying microstructural alterations, such as cytoarchitecture and myelination. Further exploration of radiality, particularly its region-specific patterns, holds promise for deepening our understanding of the brain’s structural and functional changes over the lifespan. This study builds a foundation for microstructural research of cortex alterations in development/aging, injury, and disease, providing a baseline for future research on brain health across the lifespan.

## Data Code and Availability

The full segmentation pipeline will be made available upon publication on the following GitHub page (https://github.com/grahamlittlephd/MicroBrain). For download links and data access, please email the Principal Investigator, Christian Beaulieu at christian.beaulieu@ualberta.ca.

## Supplementary Material

Supplementary Material
