# Supplementary material for: High resolution diffusion tensor imaging of the human cortex reveals non-linear trajectories over the healthy lifespan
Source: Imaging Neurosci (Camb). 2025 Aug 20;3:IMAG.a.115. doi: 10.1162/IMAG.a.115 (PMC12368611; doi:10.1162/IMAG.a.115)
Supplement: Supplementary Material [file IMAG.a.115_supp.pdf]

Supplementary Figures

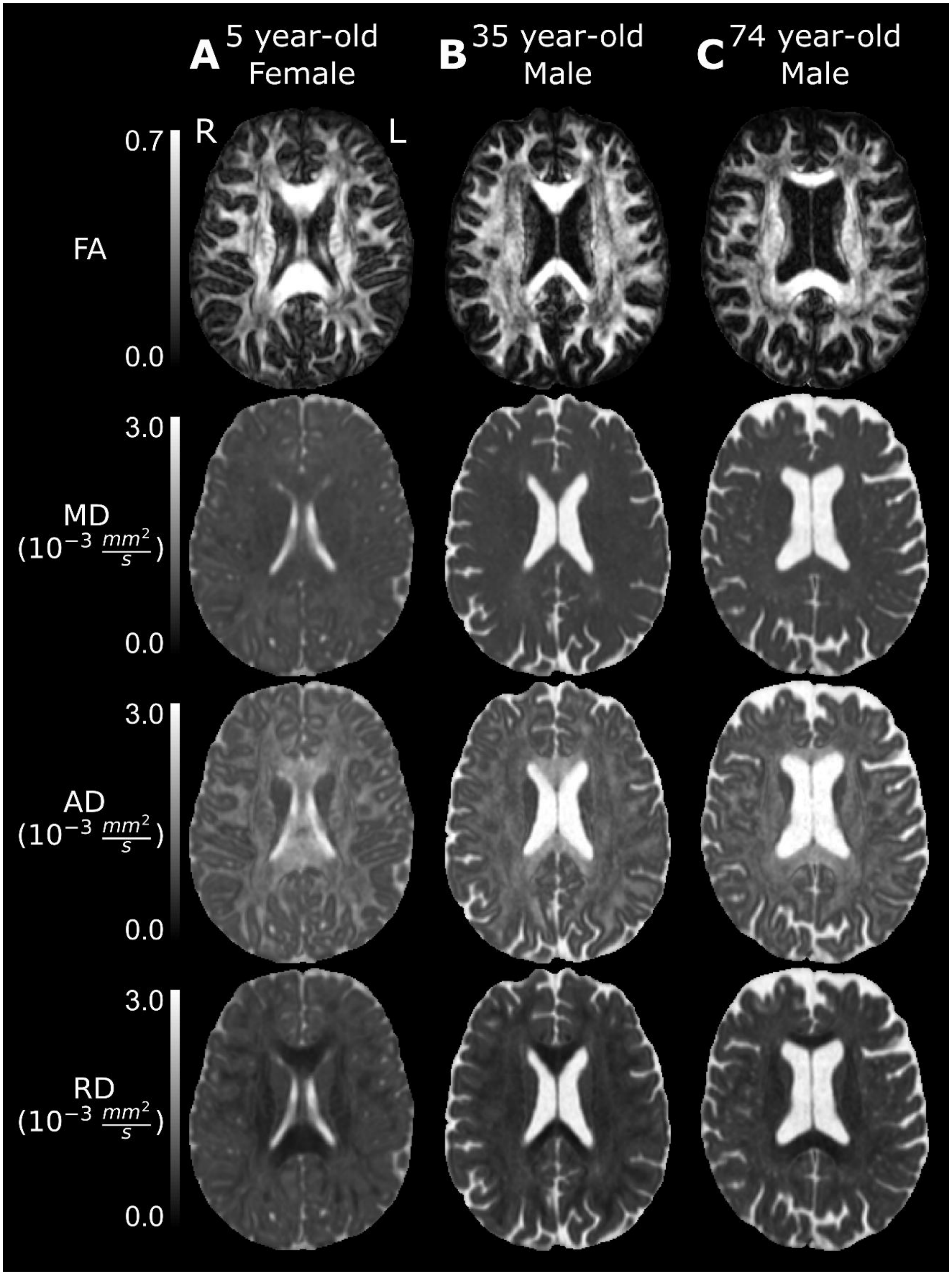

Supplementary Figure 1. Examples of 1.5 mm isotropic DTI map quality for a single axial slice of fractional anisotropy (FA) and mean (MD), axial (AD) and radial diffusivities (RD) from the same three participants with different ages shown in Figure 2.
